# Revision ACL reconstruction using quadriceps, hamstring and patellar tendon autografts leads to similar functional outcomes but hamstring graft has a higher tendency of graft failure

**DOI:** 10.1007/s00167-022-07200-2

**Published:** 2022-10-20

**Authors:** Amit Meena, Luca Farinelli, Christian Hoser, Elisabeth Abermann, Akshya Raj, Caroline Hepperger, Mirco Herbort, Christian Fink

**Affiliations:** 1 Gelenkpunkt-Sports and Joint Surgery, FIFA Medical Centre of Excellence, Olympiastraße 39, 6020 Innsbruck, Austria; 2 Research Unit for Orthopaedic Sports Medicine and Injury Prevention (OSMI), Medical Informatics and Technology, Private University for Health Sciences, Innsbruck, Austria; 3 Clinical Orthopaedics, Department of Clinical and Molecular Sciences, Università Politecnica Delle Marche, Ancona, Italy; 4 Central Institute of Orthopaedics, Vardhman Mahavir Medical College and Safdarjung Hospital, New Delhi, 110029 India; 5 OCM Clinic, Munich, Germany

**Keywords:** ACL, Anterior cruciate ligament, Revision, Quadriceps tendon, Hamstring tendon, Patellar tendon, QT, HT, BPTB, Autograft, Functional outcome, Graft failure

## Abstract

**Purpose:**

The purpose of this study was to evaluate the differences in the patient-reported functional outcomes, and graft failure in revision ACL reconstruction using quadriceps tendon (QT), Hamstring tendon (HT) and bone-patellar tendon-bone (BPTB) autografts.

**Methods:**

Between 2010 and 2020, 97 patients who underwent revision ACL reconstruction (40 patients received a QT, 26 an HT and 31 a BPTB graft) met the inclusion criteria. Pre-injury and at 2-year postoperatively patients were evaluated for patient-reported functional outcomes; Lysholm knee score, Tegner activity level and VAS (visual analogue scale) for pain; and graft failure. Patient-reported outcomes and graft failure were compared between the QT, HT and BPTB groups. The patients with graft failure were not included for outcome analysis at 2-years of follow-up.

**Results:**

All three revision groups with QT, HT and BPTB autograft did not differ significantly in terms of age, sex, time from injury to surgery, concomitant injuries and single-stage or double-stage procedures (n.s.). No significant difference was found in the pre-injury patient-reported outcome; Lysholm knee score, Tegner activity and VAS for pain (n.s.) between the three groups. At the 2-year follow-up functional outcomes improved in all three groups and all the patients returned to pre-injury activity level; however, no significant difference was found in functional outcomes at the 2-year follow-up between the three groups (n.s.). Graft failure occurred in 4 (10%), 5 (19%) and 3 (10%) patients of QT, HT and BPTB groups, respectively. However, the rate of failure did not differ significantly between groups.

**Conclusion:**

All three autografts (QT, HT and BPTB) demonstrated satisfactory patient-reported outcomes in revision ACL reconstruction. Compared with QT and BPTB grafts, HT graft showed a higher tendency for failure rates. With the increasing incidence of revision ACL reconstruction, surgeons should be aware of all the available graft options. The findings of this study will assist the surgeons in the graft selection for revision ACL reconstruction.

**Level of evidence:**

Level III.

## Introduction

Various patient-related factors such as age, sex, and tibial slope; surgeon-related factors such as graft size, graft type, and tunnel placement; other factors such as activity level, concomitant injuries, and timing of return to sport are associated with retear rates after ACL surgery [1, 7]. Graft failure is one of the most common indications for revision ACL surgery [29]. Graft choice influences the functional results and graft failure [11]. Therefore, appropriate graft selection is a crucial step of revision ACL reconstruction. In primary ACL surgery, graft choice typically depends on surgeons’ preference, while in revision surgery it depends on various factors such as the type of graft used in primary surgery, tunnel enlargement and preferred surgical technique.

Hamstring tendon (HT) and bone-patellar tendon-bone (BPTB) are the two most commonly used grafts and both grafts provided satisfactory outcomes [1, 6, 16, 29]. However, HT autograft harvest may cause sensory deficits due to injury to the ramus infrapatellaris of the saphenous nerve, compromise medial stability of the knee, and may also cause weakness of knee flexion and internal rotation [13, 14]. On the other hand, BPTB may cause anterior knee pain, limited range of movement (ROM), and increased OA (osteoarthritis) of the knee [11]. A higher failure rate was reported with HT compared to BPTB grafts [16].

In recent years, Quadriceps tendon (QT) graft has gathered enthusiasm due to lower donor site morbidity than HT and BPTB, lower failure rate than HT, greater mean cross-sectional area compared to BPTB and greater load to failure compared to other grafts [16, 20, 28]. Moreover, in a recent study better clinical outcomes were reported with QT autograft than with HT [6].

However, as far as the authors’ knowledge, no study is available that compares the functional outcome and graft failure with QT, HT and BPTB autograft in revision ACL reconstruction. Thus, the purpose of this study was to evaluate the differences in the patient-reported functional outcomes, and graft failure in revision ACL surgery using QT, HT and BPTB autografts. The hypothesis was that all three grafts would have similar functional outcomes but higher graft failure would be associated with HT compared to QT and BTBP in revision ACL reconstruction. The findings of this study will assist the surgeons in the graft selection for revision ACL reconstruction.

## Materials and methods

The study was performed at Gelenkpunkt—Sports and Joint Surgery, FIFA Medical Centre of Excellence and approved by the ethics committee of the Medical University of Innsbruck (AN2015-0050). Prospectively collected data were obtained from an ACL registry. Patients were included in the study if they fulfilled the following inclusion criteria: revision ACL reconstruction using QT, HT, BPTB autograft; age 18–55 years; and a minimum of 2-year follow-up. The exclusion criteria were: primary ACL reconstruction; contralateral knee injuries; utilization of allograft or graft other than QT, HT, BPTB; inflammatory arthritis or osteoarthritis; less than 2-year of follow-up and conditions that might interfere with the standard postoperative rehabilitation protocol.

Pre-operative CT (computed tomography) scan with 3D reconstruction was performed for all the patients to determine femoral and tibial tunnel positions, tunnel widening and tunnel convergence. The present study followed the classification for ACL revision published by Fink et al. [9]. Based on this classification type B patients (massive tunnel enlargement, anatomical placement of tunnel is not possible) were operated on in two stages (bone graft of tunnel first followed by ACL reconstruction) while, type A patients (minimal or no tunnel enlargement, tunnel completely off the anatomical location) and type C patients (tunnel slightly off the anatomical position with or without enlargement) were operated in a single stage procedure.

In most cases, a rectangular femoral tunnel was used [10]. Besides anatomical and biomechanical advantages, a rectangular tunnel allows to easier bypass an old conventional tunnel. The tibial tunnel was performed with standard round reamers. In all the patients, the surgical procedure and rehabilitation protocol were identical regardless of the graft selection.

Between 2010 and 2020, 97 patients who underwent revision ACL reconstruction (40 patients received a QT, 26 an HT and 31 a BPTB graft) met the inclusion criteria. All surgeries were performed by two fellowship-trained knee specialists. Patients were specifically asked to fill out the questionnaire considering their pre-injury state (just before failure of primary reconstructed ACL), during the first week of revision surgery for the baseline functional scores. Similarly, patients were evaluated at a 2-year follow-up for Lysholm knee score, Tegner activity level and VAS (visual analogue scale) for pain; and graft failure. Patient-reported outcomes and graft failure were compared between the QT, HT and BPTB groups. The patients with graft failure were not included for outcome analysis at 2-years of follow-up.

### Statistical analysis

A priori power analysis was performed to determine the appropriate sample size for the study. Considering an *α* level with *p* = 0.05, a power of 80%, and an effect size of 0.20 it was estimated that 63 subjects would be needed in order to detect a statistically significant difference in Lysholm knee score. The sample size calculation was performed with the use of the G-Power software (G-Power version 3.1, Düsseldorf, Germany).

Data were retrieved and organized using an Excel sheet (Microsoft, Redmond, WA, USA). Categorical variables were expressed in numbers and percentages (%). Continuous variables were expressed by average and standard deviation (SD). The normal distribution of variables was verified through Shapiro–Wilk test. Variables were not normally distributed therefore; nonparametric tests were used for the comparison of variables. Specifically, the Mann–Whitney test was used for unpaired samples, when analyzing the variables between pre-injury level and follow-up in each group. The Kruskal–Wallis test was used to analyze continuous variables between groups. The chi-square statistic test was used to determine differences in nominal data between groups, with Yates correction applied when the frequency of observations was below 5. A *p* value less than 0.05 was indicative of statistically significant differences. The statistical analysis was performed with XLSTAT (Addinsoft SARL) software packages.

## Results

Demographic details and characteristics of the study population can be found in (Table 1). All three revision groups with QT, HT and BPTB autograft did not differ significantly in terms of age, sex, time from injury to surgery, concomitant injuries and single-stage or double-stage procedures (n.s.). QT, HT and BPTB were used as primary grafts in 40, 47 and 10 patients, respectively. No significant difference was found in the pre-injury patient-reported outcome; Lysholm knee score, Tegner activity and VAS for pain (n.s.) (Table 2).

**Table 1:** Demographics data and concomitant injuries

Type of graft	QT (*n* = 40)	HT (*n* = 26)	BPTB (*n* = 31)	*p* value
Age	37.6 ± 10.5	36.2 ± 10.5	32.6 ± 8.3	n.s.
Sex ratio (male/female)	25/15	19/7	16/15	n.s.
Single stage/double stage	36/4	23/3	30/1	n.s.
Days from injury to surgery	43 ± 77	21 ± 22	23 ± 32	n.s.
Isolated revision ACL surgery	19 (47.5%)	16 (61.5%)	13 (41.9%)	n.s.
Complex revision ACL surgery	21 (52.5%)	10 (38.5%)	18 (58.1%)	
Meniscal lesion	20 (50%)	9 (34.6%)	18 (58.1%)	n.s.
Medial	10 (25%)	5 (19.2%)	8 (25.8%)	n.s.
Partial meniscectomy	7 (17.5%)	3 (11.5%)	3 (9.7%)	n.s.
Repair	3 (7.5%)	2 (7.7%)	5 (16.1%)	n.s.
Lateral	10 (25%)	4 (15.4%)	10 (32.3%)	n.s.
Partial meniscectomy	6 (15%)	1 (3.8%)	7 (22.6%)	n.s.
Repair	4 (10%)	3 (11.5%)	3 (9.7%)	n.s.
Cartilage injuries	4 (10%)	2 (7.7%)	5 (16.1%)	n.s.
MCL injuries	1 (2.5%)	1 (3.8%)	3 (9.7%)	n.s.
Graft primary ACL surgery				
QT, *N* (%)	6 (15%)	11 (42.3%)	23 (74.2%)	
HT, *N* (%)	32 (80%)	9 (34.6%)	6 (19.4%)	
BPTB, *N* (%)	2 (5%)	6 (23.1%)	2 (6.5%)	

Data are means ± standard deviation (SD). Comparison made using Kruskal–Wallis test for continuous variables; Chi-square test for categorical variables

*HT* hamstring tendon, *QT* quadriceps tendon, *BPTB* bone patella tendon bone, *n.s.* non-significant

**Table 2:** Pre-injury and 2-year post-operative subjective outcome parameters and graft failure

Pre-injury PROMs	QT (*n* = 40)	HT (*n* = 26)	BPTB (*n* = 31)	*p* value
Lysholm knee score^a^	80.6 ± 17.7	78.2 ± 20.7	73.4 ± 23.4	n.s
Tegner activity^b^	6 (2, 6–8)	6.5(2.8, 5–7.8)	7 (2, 6–8)	n.s
VAS^a^	1.2 ± 2.0	2.1 ± 2.7	2.4 ± 2.6	n.s

2-years FU PROMs^c^	QT (*n* = 36)	HT (*n* = 21)	BPTB (*n* = 28)	*p *value
Lysholm knee score^a^	89.4 ± 9.6	86.0 ± 10.3	92.1 ± 7.8	n.s
Tegner activity^b^	6 (0.3, 6–6.3)	6 (3.5, 4.3–7.8)	7 (2, 6–8)	n.s
VAS^a^	0.9 ± 1.1	1.1 ± 1.5	0.7 ± 0.8	n.s
Graft failure	4 (10%)	5 (19.2%)	3 (10%)	n.s

Comparison made using Kruskal–Wallis test for continuous variables; Chi-square test for categorical variables

*PROMs* Patients related outcomes measurements, *FU* follow-up

^a^Data are expressed as mean ± standard deviation

^b^Data are expressed as median and interquartile range

^c^As concern clinical outcomes at 2-year follow-up, patients with graft failure were excluded for analysis

**Fig. 1:**
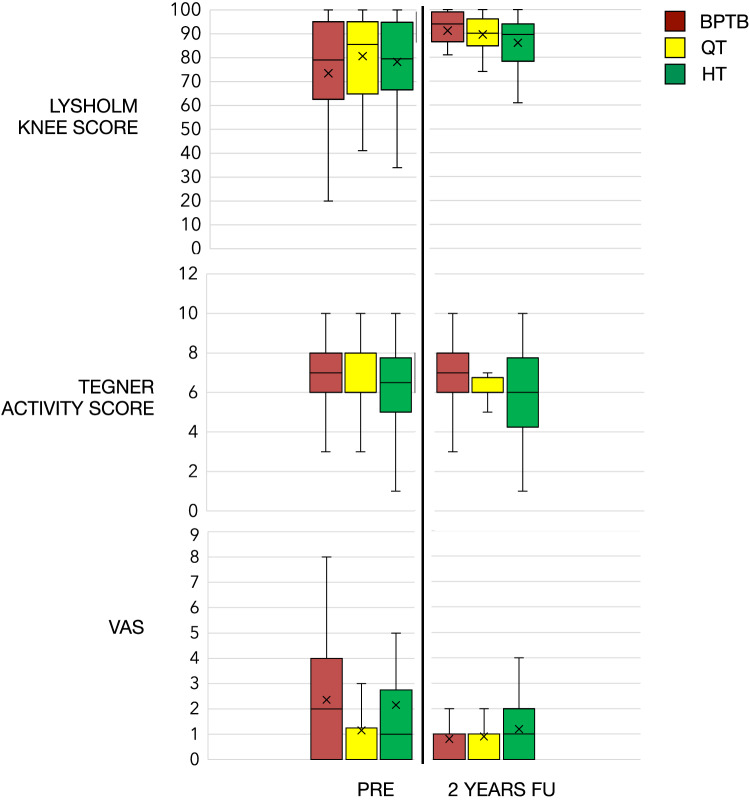
Patient-reported outcomes measures. *BPTB* Bone patellar tendon bone, *QT* quadriceps tendon, *HT* hamstring tendon. X: mean

The mean postoperative Lysholm knee score was 89.4 ± 9.6, 85.9 ± 10.3 and 92.1 ± 7.8 in QT, HT and BPTB groups respectively. The mean Lysholm score and VAS score improves respectively from a pre-injury value of 73.4 ± 23.4 and 2.4 ± 2.6 to 92.1 ± 7.8 and 0.7 ± 0.8 in the BPTB group (*p* = 0.01 and *p* = 0.04). Although improvement was noted in Lysholm score in either QT and HT groups, it did not reach statistical significance at a 2-year follow-up (n.s.) (Table 3 and Fig. 1). No significant difference was noted at the 2-year follow-up between the three groups for Lysholm, Tegner activity score and VAS (n.s.).

**Table 3:** Statistics of PROMs

PROMs	*p* value^a^
QT (*n* = 40 baseline, *n* = 36 follow-up)	
Lysholm knee score	n.s
Tegner activity	n.s
VAS	n.s
HT (*n* = 26 baseline, *n* = 21 follow-up)	
Lysholm knee score	n.s
Tegner activity	n.s
VAS	n.s
BPTB (*n* = 31baseline, *n* = 28 follow-up)	
Lysholm knee score	**0.01**
Tegner activity	n.s
VAS	**0.04**

Bold: significant difference (*p* < 0.05). Analysis performed by Mann–Whitney test for unpaired samples. Patients with graft failure were excluded for analysis

*QT* quadriceps tendon, *HT* hamstring tendon, *BPTB* bone patellar tendon bone

^a^Comparison between baseline and follow-up values

Tegner activity level increased in all three groups and all the patients returned to pre-injury activity level; however, no significant difference was found in Tegner activity between pre-injury and follow-up values in all groups (n.s.). Similarly, VAS for pain also improved and reach to pre-injury level in all three groups. No significant difference was noted in VAS at 2-year compared to the pre-injury level (n.s.) in QT and HT groups. However, in BPTB groups VAS improved significantly at 2-year follow-up compared to pre-injury level. Graft failure occurred in 4 (10%), 5 (19.2%) and 3 (9.7%) patients of QT, HT and BPTB groups respectively. However, the rate of failure did not differ significantly between groups (n.s).

## Discussion

The most important findings of this study were that after revision of ACL reconstruction satisfactory improvement was noted for patient-reported functional outcomes (Lysholm knee score, Tegner activity level and VAS for pain score) in all the groups. No difference was found in functional outcomes between the three groups at the final follow-up. All the patients returned to the pre-injury activity level. A tendency for higher graft failure was noted with HT (19.2%) than QT (10%) or BPTB (10%) grafts. To the best of the author’s knowledge, this is the first study comparing all three autografts; QT, HT and BPTB for revision ACL reconstruction.

The incidence of ACL reconstruction is increasing over the past few decades. Excellent results after primary ACL reconstruction allow the patients to participate in highly demanding activities such as jumping, cutting, deceleration, and pivoting sports [5]. These activities substantially increased the risk of retear, therefore, the need for revision surgery has also increased over time [7].

In the present study, the Lysholm, Tegner activity levels and VAS for pain scores improved and reached pre-injury levels in all three groups. No significant difference was noted in patient-reported outcomes between the three groups at a 2-year follow-up. Mouarbes et al. [19] noted no significant difference in functional outcomes at the final follow-up between the three groups. Runner et al. [24] found comparable patient-reported outcomes in the primary QT and HT groups. Similarly, in another study, no significant difference was noted in patient-reported outcomes between the primary HT and QT ACL reconstruction group [25]. Lind et al. [15] in their randomized control trial noted no difference in subjective patient outcomes between HT and QT graft groups but they reported significantly less donor site pain in the QT group. Functional outcomes are also comparable between QT and HT autograft in revision ACL reconstruction. In their revision ACL reconstruction, Barié et al. found comparable functional outcomes with QT and HT autograft [1]. Similarly, in another revision study, Häner et al. [12] also found comparable results with QT and HT autograft. Improvements in patient-reported outcomes are comparable to previous studies.

Interestingly, a significant improvement from pre-injury to final follow-up was seen in the BPTB group. In their recent study, Yumashev et al. [29] also found a significantly higher Lysholm score in the BPTB group than HT group for revision ACL reconstruction. One of the possible reasons for significant improvement in Lysholm and VAS score from pre-injury level to final follow-up in the BPTB group was comparatively younger age patients in the BPTB (32.6 ± 8.3) compared to QT (37.6 ± 10.5) and HT group (36.2 ± 10.5).

The choice of graft in revision ACL reconstruction may be limited based on previously used grafts and it depends on various factors such as age, level of sports activities, previous tunnel position, tunnel enlargement, graft used in primary ACL surgery and surgeon’s preference [2, 21]. Graft choice is especially relevant in type C revision ACL, considering the fact that a single-stage revision procedure is possible with a thicker graft diameter [9]. BPTB and QT autografts provide a thicker diameter than HT autografts, therefore, in this situation use of QT and BPTB is preferable.

Graft choice influences graft failure [11]. Multicentre ACL revision study (MARS) found that in revision ACL reconstruction re-rupture is 2.78 times less likely with autografts than with allografts [17]. Similarly, various meta-analyses also reported lower graft failure with autografts than with allografts [4, 8, 22]. Therefore, the use of autografts is especially recommended in young highly demanding patients. Considering these facts, the present study used only autografts for revision ACL reconstruction. However, the graft choice between QT, HT and BPTB autografts remains widely debated in surgical practice.

The current study found a higher tendency of graft failure with HT than with QT and BPTB autograft, although the graft failure rate was not significant between the 3 groups. Both QT and BPTB have a higher graft diameter and strength compared to an HT graft which may explain the higher failure rate of HT grafts [22]. In their systematic review, Conte et al. [3] found that if the size of HT graft is equal to or less than 8 mm, then the relative risk of failure increase by 6.8 times. A recent systematic review of registry data including Danish, Norwegian and Kaiser Permanente (KP) registries found a higher failure of HT grafts compared with BPTB grafts [22]. Similarly in another large cohort meta-analysis graft failure was higher in the HT group [26]. Eggeling et al. [6] in their recent revision ACL study, compared graft failure between QT and HT and found higher graft failure in HT (17.4%) than in QT (2.3%). These findings are in accordance with the current study.

QT autografts are used far less commonly than HT and BPTB grafts [18]. One of the major factors responsible for its lesser use is historical harvesting techniques, where extensive dissection of extensor apparatus leads to quadriceps weakness, moreover, graft harvested by older techniques was biomechanically weaker and associated with residual rotatory knee laxity [27]. But, improvements in harvest techniques, allow the surgeon to reliably yield a robust volume of QT graft without hampering the quadriceps strength and very less donor site morbidity. Recent studies compared the biomechanical properties of QT and BPTB autograft and found superior results with QT compared to BPTB [16, 20, 28]. Therefore, in recent times QT autograft increasing in popularity for revision ACL reconstruction. Winkler et al. found that the use of QT autograft for revision ACL increased significantly (49% vs. 18%, *p* < 0.001) in 2015–2020 compared to 2010–2014.

In the current study, graft failure was similar in QT and BPTB groups. In their meta-analysis, Riaz et al. [23] reported comparable graft survival and joint stability with QT and BPTB grafts but lower donor site morbidity with QT graft. Mouarbes et al. [20] confirmed these findings in their recent meta-analysis with 2856 patients and found that graft survival is comparable with QT and BPTB grafts with lesser pain at the graft harvest site in the QT group than in the BPTB group. These meta-analyses with a large patient cohort suggest that QT and BPTB autografts are comparable for graft failure. However, both meta-analyses included studies with primary ACL reconstruction. To the best of the author’s knowledge, no study is available for revision ACL reconstruction comparing QT and BPTB graft, therefore, comparison of QT and BPTB autograft for revision ACL reconstruction with previous studies is not possible.

There are a few limitations of the present study. The first and the most important limitation is the small sample size, that underpowered to identify any difference in functional outcomes and graft failure. Revision ACL reconstruction is a less frequently performed procedure than primary reconstruction. Second, this was a retrospective analysis of patient-reported subjective outcome measures; however, all data were collected prospectively. A prospective study considering objective scores along with subjective scores should be conducted.

The clinical relevance of the present study lies in the fact that the incidence of revision ACL reconstruction is increasing and surgeons should be aware of all the available graft options. QT autograft is the least studied and least used graft compared to other grafts, especially for revision ACL reconstruction. Many surgeons do not even consider the QT as a possible graft option when discussing with the patients. Promising clinical results continues to emerge concerning the viability of QT autograft in revision ACL reconstruction. Based upon the findings of this study surgeon can counsel and advise the patient regarding the graft choice for revision ACL reconstruction.

## Conclusion

All three autografts (QT, HT and BPTB) demonstrated satisfactory patient-reported outcomes in revision ACL reconstruction. Compared with QT and BPTB grafts, HT graft showed a higher tendency for failure rates. With the increasing incidence of revision ACL reconstruction, surgeons should be aware of all the available graft options. The findings of this study will assist the surgeons in the graft selection for revision ACL reconstruction.
